# Alprostadil vs. isosorbide dinitrate in ameliorating angina episodes in patients with coronary slow flow phenomenon: A randomized controlled trial

**DOI:** 10.3389/fcvm.2022.965364

**Published:** 2022-09-08

**Authors:** Weifeng Zhang, Jinjie Dai, Lan Shen, Yue Jiang, Xiaowen Zheng, Ke Xu, Xiaoxiao Yang, Xiaolei Wang, Ziyong Hao, Yu Zhao, Dong Wang, Lisheng Jiang, Xingbiao Qiu, Linghong Shen, Ben He

**Affiliations:** ^1^Department of Cardiology, Shanghai Chest Hospital, Shanghai Jiao Tong University, Shanghai, China; ^2^Clinical Research Center, Shanghai Jiao Tong University, Shanghai, China; ^3^Department of Cardiology, The First Affiliated Hospital of Zhengzhou University, Zhengzhou, China

**Keywords:** coronary slow flow phenomenon, angina pectoris, alprostadil, treatment, outcome

## Abstract

**Background:**

The optimum therapy for coronary slow flow phenomenon (CSFP) stays debatable. This study compared the effectiveness of alprostadil with isosorbide dinitrate in alleviating angina episodes in CSFP patients.

**Methods:**

In this prospective, randomized controlled study, 102 patients with CSFP without severe coronary artery stenosis that exhibited stable angina were allocated randomly in a ratio of 1:1 to either the alprostadil group (40 μg, three times per day, *n* = 51) or the isosorbide dinitrate group (5 mg, three times per day, *n* = 51). Frequency of angina events, intensity of suffering, and the Canadian Cardiovascular Society (CCS) grading of angina pectoris were evaluated at baseline and one month after. Additionally, the Seattle Angina Questionnaire (SAQ) was assessed.

**Results:**

Baseline characteristics were comparable between the two groups. At 1-month follow-up, patients administered with alprostadil experienced fewer angina episodes [episodes per week, 1 (2) vs. 2 (2), *P* < 0.001] and less pain intensity [self-evaluated pain score, 2 (3) vs. 3 (4), *P* < 0.001] than those with isosorbide dinitrate. In the alprostadil group, 78.4% of patients were classified as CCS class I, significantly higher than the 47.1% seen in the isosorbide dinitrate group (*P* = 0.001). Furthermore, treatment of alprostadil led to a significant improvement in the SAQ score (7.09 U, 95% CI: 4.22–9.96, *P* < 0.001) compared to isosorbide dinitrate. Additionally, fewer patients suffered headaches when receiving alprostadil (7.8% vs. 19.6%, *P* = 0.084).

**Conclusion:**

Alprostadil was more effective in ameliorating angina symptoms in CSFP patients than isosorbide dinitrate.

**Clinical trial registration:**

[www.chictr.org.cn], identifier [ChiCTR2000033233].

## Introduction

The coronary slow flow phenomenon (CSFP) is characterized by the late opacification of epicardial coronary arteries in the absence of severe stenosis (1). Once regarded as a harmless angiographic curiosity without clinical or prognostic significance, it has now been considered as a vital entity associated with manifestations of myocardial ischemia, acute coronary syndrome, life-threatening arrhythmias and even sudden cardiac death over the past decades (2–5).

The pathophysiology of CSFP remains controversial. Observational data suggested that coronary microvascular dysfunction might play a most possible role in this setting (6). Meanwhile, the treatment for CSFP is also elusive due to limited evidence on optimal pharmacological approaches. Conventional vasodilators, primarily affecting large coronary vessels such as nitroglycerin, are only partially effective in alleviating angina symptoms in patients with CSFP (7, 8). Hence, medications that exert vasodilatory effects on coronary arterioles could be hypothetical choice of anti-angina treatment against CSFP.

Alprostadil, a liposomal prostaglandin E1 (PGE1), was reported to be effective in improving CSFP in patients diagnosed with acute ST-segment elevation myocardial infarction (STEMI) receiving primary percutaneous coronary intervention (PCI), based on its pharmacologic effects including dilation in coronary arterioles, inhibition of platelet aggregation, and prevention of ischemia-reperfusion injury (9). However, the potential role of alprostadil in ameliorating stable angina episodes among patients with CSFP has not been investigated.

In this prospective, randomized controlled study, we evaluated the efficacy of alprostadil in ameliorating angina episodes, as well as its discrepancy between isosorbide dinitrate in CSFP patients without severe coronary artery stenosis who suffered recurrent angina symptoms.

## Materials and methods

### Study population

**FIGURE 1:**
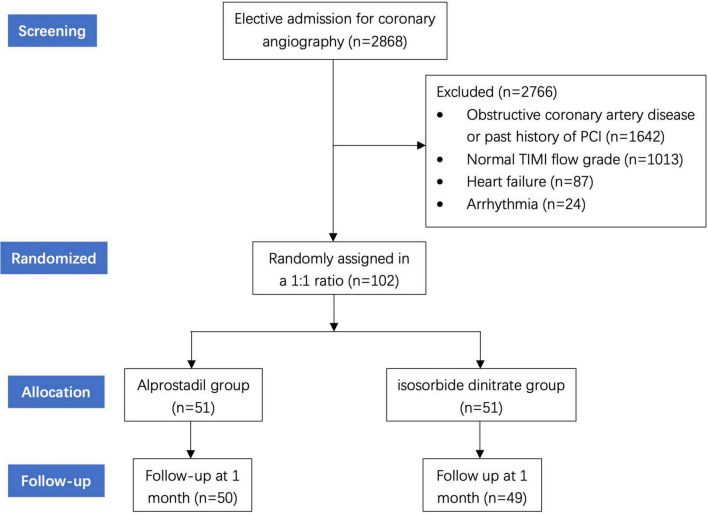
The flowchart diagram of the study.

From May 2019 to May 2021, a total of 2,868 patients presented with stable angina who received scheduled coronary angiography were admitted at the Cardiology Center of Shanghai Chest Hospital affiliated to Shanghai Jiao Tong University. Patients were included if the following criteria were met: (i) angiographically normal or near-normal (less than 30% stenosis) in all three major epicardial coronary arteries; (ii) delayed opacification in at least one major coronary arteries. Thrombolysis In Myocardial Infarction (TIMI) flow grade was used to evaluate CSFP (10), defined as TIMI grade 2 (requiring over three beats to opacify the distal artery). Exclusive criteria were as follows: (i) contraindication for the administration of alprostadil; (ii) past history of myocardial infarction, PCI or coronary artery bypass grafting (CABG); (iii) left ventricular systolic dysfunction (left ejection fraction less than 50%); (iv) sinus node disease, conduction block, frequent atrial or ventricular arrhythmia; (v) other comorbidities with prognosis less than 12 months. Among all patients, 1,642 were excluded for obstructive coronary artery disease or past history of PCI. We further excluded 1,013 patients with normal TIMI flow grade. Additional examinations revealed heart failure in 87 patients and arrhythmia in 24 patients, whom were thus not included. Therefore, 102 patients were finally enrolled in this study (Figure 1).

The Shanghai Chest Hospital’s ethics committee authorized the trial. All participants provided written informed consent before enrolling, and the procedures conformed to the standards outlined in the Declaration of Helsinki. The research was registered at *www.chictr.org.cn* (Unique identifier: ChiCTR2000033233).

### Study protocol

This study was a prospective, randomized controlled trail. Eligible patients were randomized to the alprostadil group and the isosorbide dinitrate group in a ratio of 1:1 using computerized random numbering. Patients in the alprostadil group were given standard treatment plus oral alprostadil (Beijing Tide Pharmaceutical Co., Ltd., Beijing, China; 40 μg, three times per day) for 1 month. Patients in the isosorbide dinitrate group received standard treatment plus oral isosorbide dinitrate (Fudan Forward Pharmaceutical Group, Shanghai, China; 5 mg, three times per day). Anti-platelet medication and a 3-hydroxy-3-methylglutaryl-CoA reductase inhibitor comprised the standard treatment. Other medications including angiotensin converting enzyme inhibitor (ACEI)/angiotensin II receptor blocker (ARB), β-blockers or calcium antagonists were at the cardiologists’ discretion.

Coronary angiographic procedures were conducted *via* radial or femoral access. Angiograms were obtained at 15 frames per second through 6F catheters with manual injection of low-osmolar contrast agent. Coronary arteries were accurately evaluated in multiple angulations, including the left and right anterior oblique views with cranial and caudal angulations. Blood samples were routinely collected in admission. Echocardiography was also conducted during hospitalization. The left ventricular ejection fraction (LVEF) was calculated using biplane Simpson’s method (11).

### Clinical assessment

All patients were assigned for outpatient clinic follow-up until 1 month after discharge by outpatient clinic visit. The following endpoints were measured at baseline and during the follow-up visit:

(i) Frequency of typical angina episodes per week;
(ii) Pain intensity of angina episodes evaluated by a Likert-type scale, where 0 indicated “no pain” and 10 indicated “worst pain imaginable”;
(iii) Severity of angina episodes assessed *via* the Canadian Cardiovascular Society (CCS) classification (12). In which, class I (no symptoms with ordinary activity), class II (slight limitation of ordinary activity), class III (marked limitation of ordinary activity), and class IV (symptoms at rest);

The Seattle Angina Questionnaire (SAQ) (13), a self-administered, disease-specific metric of angina, was also assessed at 1 month. It quantifies 5 domains assessing the impact of angina on health status, including patients’ physical constraints resulting from angina (9 items), the latest changes (1 item) and frequency (2 items) of their symptoms, their fulfillment of treatment (4 items), and the extent to which they regard their disease to affect quality of life (3 items). Scores are generated for each domain on a 0–100 scale, with higher scores indicating better function (14, 15).

### Statistical analysis

Means and standard deviations for normally distributed variables, or medians and interquartile ranges for non-normally distributed variables were used to express continuous variables. Categorical variables are shown as numbers and percentages (%). Across trial groups, continuous variables were analyzed using the independent t test for normally distributed values and the Mann-Whitney test for non-normally distributed values. Proportions were compared using the chi-square, and if the expected frequency < 5, the Fisher’s exact test was conducted. *P* < 0.05 was considered statistically significant. All analyses were conducted using the SPSS 23.0 software (SPSS Inc., Chicago, Illinois, United States).

## Results

### Baseline characteristics

102 patients with CSFP without severe coronary artery stenosis were randomly allocated to the alprostadil group (*n* = 51) or the isosorbide dinitrate group (*n* = 51). In the final analysis, all patients were considered. Table 1 presents baseline parameters and coronary angiographic findings. No significant difference was found in age, gender, proportions of hypertension and diabetes mellitus, levels of total cholesterol, low density lipoprotein, C-reactive protein, and LVEF between the two groups. The frequency and pain intensity of angina episodes, and distribution of the CCS classification were comparable at baseline. Based on coronary angiography findings, the number of vessels with delayed opacification was also similar between the two groups.

**TABLE 1:** Clinical characteristics and coronary angiographic findings of all patients.

Characteristics	Alprostadil (*n* = 51)	Isosorbide dinitrate (*n* = 51)	*P*-value
Age, yrs	61.6 ± 8.4	62.5 ± 9.2	0.606
Male, *n* (%)	35 (68.6%)	30 (58.8%)	0.303
Current smoking, *n* (%)	21 (41.2%)	17 (33.3%)	0.413
Hypertension, *n* (%)	22 (43.1%)	25 (49.0%)	0.551
Diabetes mellitus, *n* (%)	9 (17.6%)	11 (21.6%)	0.618
Total cholesterol, mmol/L	4.71 ± 1.06	4.54 ± 1.14	0.437
Low density lipoprotein, mmol/L	3.02 ± 0.80	2.86 ± 0.87	0.346
C-reactive protein, mg/dL	1.73 ± 2.19	1.86 ± 2.57	0.791
LVEF,%	65.2 ± 2.6	64.8 ± 3.6	0.654
Frequency of angina episodes, times per week	4 (2)	4 (2)	0.962
Pain intensity in angina episodes, 0–10/10	7 (2)	7 (2)	0.665
The CCS classification			0.780
Class I, *n* (%)	0 (0%)	0 (0%)	
Class II, *n* (%)	44 (86.3%)	43 (84.3%)	
Class III, *n* (%)	7 (13.7%)	8 (15.7%)	
Number of vessels			0.591
One, *n* (%)	20 (39.2%)	25 (49.0%)	
Two, *n* (%)	8 (15.7%)	6 (11.8%)	
Three, *n* (%)	23 (45.1%)	20 (39.2%)	
Multivessel disease, *n* (%)	31 (60.8%)	26 (51.0%)	0.319
Medications			
Anti-platelet agents, *n* (%)	51 (100%)	51 (100%)	1.000
Statins, *n* (%)	51 (100%)	51 (100%)	1.000
ACEI/ARB, *n* (%)	12 (23.5%)	14 (27.5%)	0.650
β-blockers, *n* (%)	11 (21.6%)	16 (31.4%)	0.262
Calcium antagonists, *n* (%)	15 (29.4%)	14 (27.5%)	0.826

Variables are expressed as means ± standard deviations, medians (interquartile ranges), and n (%). LVEF, left ventricular ejection fraction; CCS, Canadian Cardiovascular Society; ACEI, angiotensin converting enzyme inhibitor; ARB, angiotensin II receptor blocker.

### Efficacy of alprostadil and isosorbide dinitrate in ameliorating angina episodes

**FIGURE 2:**
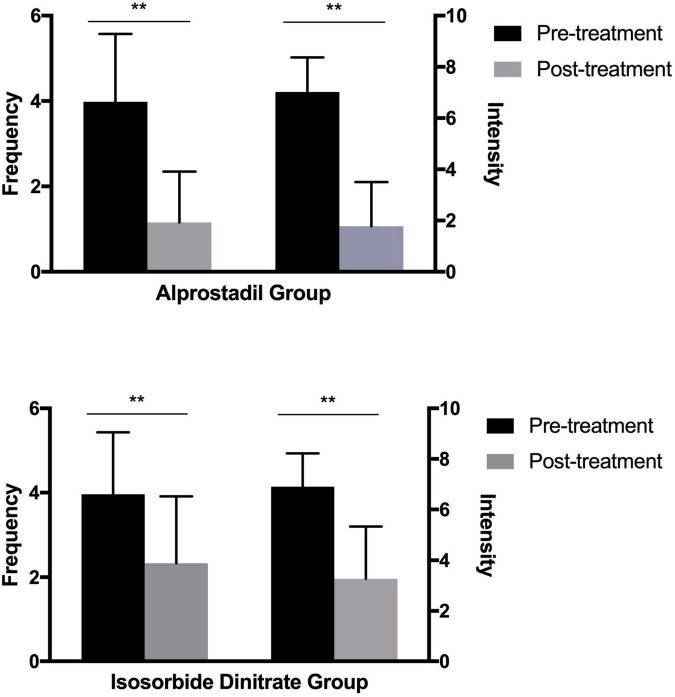
Efficacy of alprostadil and isosorbide dinitrate in the amelioration of angina frequency and intensity (medians and interquartile ranges). Statistical analysis was performed using the Mann-Whitney test. **P < 0.01.

**FIGURE 3:**
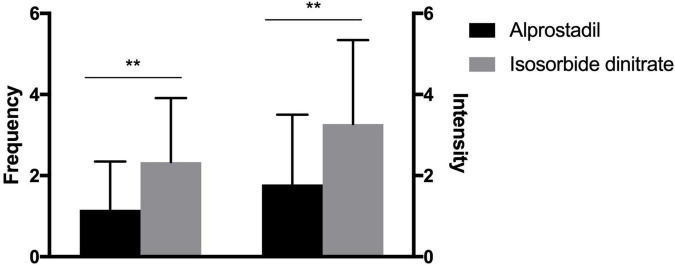
Comparison of alprostadil vs. isosorbide dinitrate in the efficacy of alleviating angina frequency and intensity (medians and interquartile ranges). Statistical analysis was performed using the Mann-Whitney test. **P < 0.01.

A significant decrease was found in both frequency and pain intensity of angina episodes [episodes per week, 4 (2) vs. 1 (2), *P* < 0.001; self-evaluated pain score, 7 (2) vs. 2 (3), *P* < 0.001; Figure 2] after 1-month treatment of alprostadil. Similar trend was also observed among patients in the isosorbide dinitrate group [episodes per week, 4 (2) vs. 2 (2), *P* < 0.001; self-evaluated pain score, 7 (2) vs. 3 (4), *P* < 0.001; Figure 2]. However, patients treated with alprostadil suffered fewer angina episodes [episodes per week, 1 (2) vs. 2 (3), *P* < 0.001] and less pain intensity [self-evaluated pain score, 2 (2) vs. 3 (4), *P* < 0.001], compared with those in the isosorbide dinitrate group (Figure 3).

**FIGURE 4:**
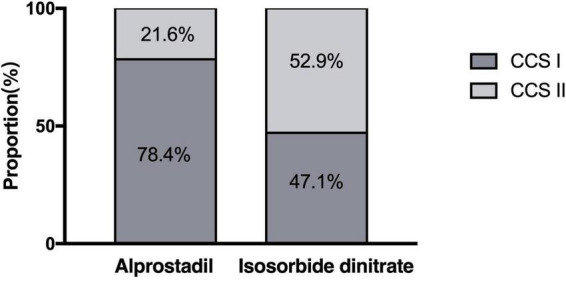
Distribution of the Canadian Cardiovascular Society (CCS) classification of angina episodes after 1-month treatment of alprostadil and isosorbide dinitrate (proportions). Statistical analysis was performed using the chi-square test.

At 1-month follow-up, a downward tendency in the CCS classification of angina episodes was observed in 88.2% (45/51) of the patients treated with alprostadil, compared with 58.8% (30/51) of those with isosorbide dinitrate (*P* = 0.001). In the alprostadil group, 78.4% of patients were classified as CCS class I after treatment, significantly higher than the 47.1% in the isosorbide dinitrate group (*P* = 0.001; Figure 4).

### The Seattle Angina Questionnaire assessment

Three patients excused themselves from evaluating SAQ score at the clinic visit. Therefore, a total of 99 patients completed the SAQ assessment at 1 month (50 in the alprostadil group, and 49 in the isosorbide dinitrate group). Administration of alprostadil induced a significant increase of 7.09 U (95%CI: 4.22–9.96, *P* < 0.001) for the SAQ score, compared with isosorbide dinitrate. Taken separately, improvement was found in all 5 domains of metric, including patients’ physical limitations (1.97 U, 95%CI: 0.67–3.26, *P* = 0.003), stability (0.65 U, 95%CI: 0.40–0.90, *P* < 0.001) and frequency (1.44 U, 95%CI: 0.79–2.09, *P* < 0.001) of angina, their satisfaction with treatment (1.77 U, 95%CI: 0.83–2.71, *P* = 0.001), and angina-related quality of life (1.26 U, 95%CI: 0.54–1.98, *P* = 0.001). The individual components of the SAQ score are presented in Table 2.

**TABLE 2:** The Seattle Angina Questionnaire of all patients assessed at 1 month.

	Alprostadil (*n* = 50)	Isosorbide dinitrate (*n* = 49)	Mean difference	95% confidential interval	*P*-value
SAQ score	92.6 ± 5.1	85.5 ± 8.7	7.09	4.22–9.96	< 0.001**
Physical limitation	43.6 ± 1.8	41.6 ± 4.2	1.97	0.67–3.26	0.003**
Angina stability	4.9 ± 0.2	4.3 ± 0.8	0.65	0.40–0.90	< 0.001**
Angina frequency	10.4 ± 1.6	9.0 ± 1.6	1.44	0.79–2.09	< 0.001**
Treatment satisfaction	19.4 ± 1.3	17.6 ± 3.0	1.77	0.83–2.71	0.001**
Quality of life	14.3 ± 1.1	13.0 ± 2.3	1.26	0.54–1.98	0.001**

SAQ, Seattle Angina Questionnaire. **P < 0.01.

### Safety of drug administration

During the study phase, neither malignant arrhythmias nor hemodynamic abnormalities were observed after administration of alprostadil or isosorbide dinitrate. 7.8% (4/51) of patients in the alprostadil group reported transient headaches, compared with 19.6% (10/51) of those in the isosorbide dinitrate group (*P* = 0.084).

## Discussion

To the best of our knowledge, this is the first study evaluating the efficacy of alprostadil in alleviating angina symptoms, and its discrepancy between isosorbide dinitrate in patients with CSFP without significant stenosis in coronary arteries. Our data demonstrated that alprostadil was more effective in reducing the frequency, pain intensity, and severity of angina episodes, thus leading to a better quality of life.

Angiographic observations of CSFP are widespread, with a reported prevalence of 1–7% in patients undergoing coronary angiography (1). It occurs most commonly in young men and smokers (16), manifested by recurrent chest pain, acute coronary syndrome, life-threatening arrhythmia, and even sudden cardiac death in this group of patients due to increased QTc dispersion (17). The pathogenic mechanisms of CSFP remains under debate. Coronary microvascular dysfunction is considered the main cause of CSFP (18). Evidence of fibromuscular hyperplasia, medial hyperplasia, myointimal proliferation, as well as edema at the electron microscopic level validated this hypothesis (19). Decreased flow-mediated dilation (FMD) of brachial coronary arteries, a mechanism reliant on endothelium, was also reported in participants with CSFP, highlighting the possibility that endothelial dysfunction contributes to CSFP etiology (20, 21). Other factors including inflammatory status (22, 23), subclinical atherosclerosis (24, 25), and geometric irregularities in major coronary arteries (26, 27) were also reported to be involved in this regard.

The complexity and uncertainty in pathogenic mechanisms of CSFP result in limited evidence on pertinent pharmacological approaches. Previously, a small-scale study suggested that mibefradil, a T-type calcium channel blocker, was significantly effective in reducing the frequency of angina episodes and the need for rescue consumption of sublingual nitrate as well. The efficacy of mibefradil might be ascribed to the selective blockade of T-type channels, which are abundant in coronary branches of low diameter (less than 200 μm) (28). Besides, similar findings have also been acquired from dipyridamole and nicorandil, which both showed a promising influence in ameliorating the frequency and pain intensity of angina episodes due to their positive effect on microvascular function (29, 30).

Alprostadil, a PGE1, was reported to play a beneficial role in improving the epicardial and myocardial perfusion in STEMI patients undergoing primary PCI with post-procedure CSFP (9). Benefiting from its dosage form, alprostadil can act directly upon the targeted lesion with limited systemic adverse consequence. In this study, we found that oral administration of alprostadil induced a greater reduction in the frequency and pain intensity of angina episodes during the follow-up period, compared with isosorbide dinitrate. The profitable effect of alprostadil might be attributed to its pharmacologic effects in dilating coronary arterioles, relieving microvascular spasm, decreasing platelet aggregation, and inhibiting inflammation (31). The difference in angina frequency and pain intensity was consistent with the superior improvement in the CCS classification of angina episodes among patients treated with alprostadil at 1-month follow-up. In addition, the overall treatment effect of alprostadil was reflected by a relative increase of over 7 U in the SAQ score compared with isosorbide dinitrate. This was driven by reduced angina limitation, enhanced angina stability, less angina frequency, better treatment fulfillment, and improved quality of life related to angina. In a word, we complemented the previous research, demonstrating that alprostadil was also effective in the alleviation of angina episodes and provision for a better life quality in CSFP patients without severe coronary artery stenosis (32).

Furthermore, over the study phase, 4 patients taking alprostadil suffered transient headaches largely relieved by the administration of non-steroidal anti-inflammatory drugs. The incidence of this most frequent side effect was lower compared with isosorbide dinitrate group, suggesting oral use of alprostadil a more effective and well-tolerant treatment for CSFP. In addition, no hemorrhagic events were observed in this study. Previous study suggested that PGE1 produced no significant change in platelet function (either bleeding time or platelet adhesiveness) in healthy volunteers (33). Besides, though considered to reduce platelet reactivity with combined use of dual antiplatelet therapy (34), the administration of alprostadil appears to be safe in patients treated with peroral antithrombotics (35). However, future studies are still necessary to investigate the potential risk of bleeding especially in the long-term period. Finally, no patient reported irregular erection in this study, possibly due to personal or conceal consideration. Intraurethral and topical use of alprostadil have been proved to be effective in men with erectile dysfunction with tolerable side effects (36, 37), both recommended as second-line therapy from the Fourth International Consultation for Sexual Medicine (ICSM 2015) (38). Therefore, it cannot be neglected that oral administration of alprostadil might also cause erection in male patients even in the absence of sexual arousal, which could be regarded as a beneficial or unfavorable side effect. This potential phenomenon should be informed to the patients beforehand, and deserves further investigation in the future follow-up period.

Note that the reduction in the frequency and pain intensity of angina episodes was also observed in patients treated with isosorbide dinitrate. This phenomenon was partially consisted with the previous study (30). Though thought to have limited vasodilatory effects on coronary arterioles, traditional anti-ischemic drugs such as nitrates are still recommended to treat anginal attacks despite its unreliable effect (39). Moreover, since the anginal complaints were ameliorated with administration of either alprostadil or isosorbide dinitrate in this trial, future studies are encouraged to assess the possibility of a combined drug approach on alleviating angina episodes in patients with CSFP, which are hypothetically effective in dilating both large and small coronary vessels. Another possible factor for the positive result in the isosorbide dinitrate group might be ascribed to the standard treatment of statins, which has been shown beneficial for patients with CSFP due to their pleiotropic effect including endothelial function protection and anti-inflammatory action (40–42).

## Study limitations

We noted that this study has several limitations. First, this was a single-blinded and relatively small-scale study carried out by a single center. We acknowledged that a double-blinded trial would be more convincing in the comparison of therapeutic effect with alprostadil and isosorbide dinitrate. However, the discrepancy in the appearance of tablets between these two drugs led us to adopt the form of single-blinded study, in which isosorbide dinitrate (Fudan Forward Pharmaceutical Group, Shanghai, China) is white in color, while alprostadil (Beijing Tide Pharmaceutical Co., Ltd., Beijing, China) appears to be yellow, both round in shape. The doctors were hence able to realize the allocation of a patient when he/she was given pills or at outpatient clinic visit. In addition, though observed a superior efficacy over isosorbide dinitrate, this trial might not be sufficiently designed to detect uncommon side effects associated with alprostadil administration such as skin rashes, facial flushing or peptic ulcers. A double-blinded trial with a larger sample of patients from multiple centers would make it more reliable and objective in terms of efficacy and safety. Second, we were unable to elucidate the instant effect of alprostadil on coronary flow rate in patients with CSFP. Future studies are necessary to assess the angiographic effect of alprostadil *via* intracoronary administration during the coronary angiography procedures. Third, subjective bias might exist in this study, as the four end points assessed are all subjective indicators. Additional objective measurements, such as treadmill test or other exercise tolerance examination, could be included to further verify the superior effect of alprostadil on CSFP in the future studies. Fourth, the evaluation of angina episodes was conducted at baseline and 1 month after discharge. Since people tend to have a deeper impression of what happened in last few days, it would be more reasonable to collect the information by telephone every week so as to avoid recall bias. Last but not least, we expected a low rate of hard endpoints (e.g., death or acute coronary syndrome) in this relatively small-scale study. Therefore, long-term prognosis was not assessed. However, it was perceivable that patients with CSFP were likely to benefit from persistent treatment with alprostadil. It deserved further investigation in these patients for long-term follow-up.

## Conclusion

Alprostadil was more effective in ameliorating angina symptoms in CSFP patients than isosorbide dinitrate.

## Data availability statement

The raw data supporting the conclusions of this article will be made available by the authors, without undue reservation.

## Ethics statement

The studies involving human participants were reviewed and approved by the Ethic Committee of Shanghai Chest Hospital. The patients/participants provided their written informed consent to participate in this study.

## Author contributions

WZ, JD, LHS, and BH: study conception, design, and writing. LS: data analysis and interpretation. YJ, XZ, KX, XY, XW, ZH, YZ, and DW: execution and data collection. LJ and XQ: supervision. All authors contributed to the article and approved the submitted version.
